# Validation of the Arabic version of the general medication adherence scale in patients with type 2 diabetes mellitus in Jordan

**DOI:** 10.3389/fphar.2023.1194672

**Published:** 2023-09-20

**Authors:** Md. Ashraful Islam, Faris El-Dahiyat, Ahmed Nouri, Qais Alefan, Atta Abbas Naqvi

**Affiliations:** ^1^ Department of Pharmacy Practice, College of Clinical Pharmacy, Imam Abdulrahman Bin Faisal University, Dammam, Saudi Arabia; ^2^ Clinical Pharmacy Program, College of Pharmacy, Al Ain University, Al Ain, United Arab Emirates; ^3^ Medical Faculty, Institute of Anatomy II, University Hospital Düsseldorf, Heinrich-Heine-University, Düsseldorf, Germany; ^4^ Department of Clinical Pharmacy, Faculty of Pharmacy, Jordan University of Science and Technology, Irbid, Jordan; ^5^ Department of Pharmacy Practice, School of Pharmacy, University of Reading, Whiteknights Campus, Reading, United Kingdom

**Keywords:** validation studies, patient compliance, medication adherence, diabetes, Jordan

## Abstract

**Background:** Medication adherence is a major challenge for patients with diabetes. Adherence rates are often low, and this can lead to poor glycaemic control and increased risk of complications. There are a number of tools available to measure medication adherence, but few have been validated in Arabic-speaking populations.

**Aim:** This study aimed to validate the Arabic version of the General Medication Adherence Scale in patients with type 2 diabetes in Jordan.

**Methods:** A cross-sectional study was conducted for 3 months among patients attending diabetes mellitus outpatient clinic in Irbid, Jordan. The validation procedure included confirmatory factor analysis (CFA) and equation modelling (SEM). Fit indices, namely, goodness of fit index (GFI), Tucker Lewis index (TLI), comparative fit index (CFI), and root mean square error of approximation (RMSEA) were observed. Corrected item-total correlation (ITC) was reported. Reliability was assessed using Cronbach’s alpha (α) and α value based on item deletion was also carried out. Intraclass correlation coefficient (ICC) was reported. Data were analyzed using IBM SPSS v23 and IBM AMOS v25.

**Results:** Data from 119 participants were gathered. The mean adherence score was 27.5 (±6) ranging from 6 to 33. More than half of the patients were adherent to their therapy (n = 79, 66.4%). The reliability of the scale (n = 11) was 0.907, and ICC ranged from 0.880—0.930: 95% CI. The following values were observed in CFA; χ^2^ = 62.158, df = 41, χ^2^/df = 1.516, GFI = 0.913, AGFI = 0.860, TLI = 0.960, CFI = 0.971 and RMSEA = 0.066. A total of 10 out of 11 items had corrected ITC >0.5. The α remained between 0.89–0.92 during item deletion.

**Conclusion:** The results obtained in this study suggest that the scale is valid and reliable in measuring adherence to medications in the studied sample of patients with diabetes. This scale can be used by clinicians in Jordan to assess adherence and may further aide in evaluating interventions to improve adherence rates in persons with type 2 diabetes.

## 1 Introduction

Chronic illnesses are usually prolonged illnesses that are managed throughout the course of a patient’s life and are not often completely cured (Dowrick et al., 2005). They may require either life-long or long-term medication therapy and the success of long-term treatments depends on proper adherence to the medication therapy regimen (World Health Organization, 2003; Dowrick et al., 2005; Naqvi et al., 2019). According to the Institute for Health Metrics and Evaluation (IHME), chronic illnesses remain the leading cause of death alone, as well as death and disability combined. Seven chronic diseases are listed among the top 10 causes of death among the Jordanian population (Institute of Health Metrics and Evaluation IHME, 2020).

One of the most common reasons for failing to achieve treatment outcomes by patients with chronic illnesses is non-adherence to medication therapy (Naqvi et al., 2020a). The World Health Organization (WHO) defines adherence as the as the degree to which a person’s medicine taking behaviour, lifestyle changes, and dietary habits align with the recommendations given by a healthcare professional (World Health Organization, 2003). Non-adherence to pharmacotherapy may result in disease complications, prolonged disease condition, increased risk for hospitalization, risk of disability and death; all of these may further exacerbate the healthcare costs (Naqvi et al., 2020a; Al-Qasem et al., 2011; Iuga and McGuire, 2014). Non-adherence continues to prevail in developing countries as evidence highlights that only 50% of patients with chronic illnesses adhere to the treatment (World Health Organization, 2003). A review by Al-Qasem et al. highlighted that the practice of non-adherence was prevalent among patients in the Middle Eastern countries. The rate of non-adherence to medications was reported to be between 1.4% and 88% (Al-Qasem et al., 2011).

In Jordan, medication non-adherence is a serious healthcare issue where most patients are non-adherent (Al-Sahouri et al., 2019). For example, in a study that evaluated the effectiveness of home medication management review on medication adherence in Jordanian patients, most patients had medium adherence at baseline (Al‐Qudah et al., 2018). Moreover, in another study among patients with chronic ailments in Jordan, it was reported that 46.1% were non-adherent (Basheti et al., 2016). Focusing on type 2 diabetes (T2DM), it was reported that slightly less than half of the surveyed T2DM patients (46.5%) had moderate adherence while 12.2% had low adherence to their anti-diabetic medications (Al-Qerem et al., 2021).

In the past, adherence to medication among patients in Jordan has been documented using the Arabic versions of the 8–item Morisky’s Medication Adherence Scale (MMAS—8), Beliefs about Medication Questionnaire (BMQ), and Medication Adherence Report Scale (MARS) (Awwad et al., 2015; Basheti et al., 2016; Alsous et al., 2017; Al‐Qudah et al., 2018; Al-Qerem et al., 2021; Al-Qerem et al., 2022). Recently, the Arabic version of the General Medication Adherence Scale (GMAS) was validated in patients with chronic diseases in Saudi Arabia and in patients with diabetes in Sudan and Morocco (Al-Qasem et al., 2011; Mahmoud et al., 2021; Maryem et al., 2023). This scale considers non-adherence due to the cost of medications which adds a financial aspect in reporting medication adherence. The concurrent validity of the Arabic version has been evaluated by correlating the adherence scores obtained by GMAS—AR with the ones obtained by the Arabic versions of Adherence to Refills and Medications Scale (ARMS) and MARS in Saudi Arabia (Islam et al., 2021). Thus, validation of this scale is a prerequisite to enable clinicians to use the scale in Jordan.

## 2 Objective

The aim of this study was to validate the Arabic version of the General Medication Adherence Scale (GMAS—AR) in Jordanian patients with type 2 diabetes mellitus (T2DM).

## 3 Methods

### 3.1 Study design

A cross-sectional study was conducted from August to October 2018 in an outpatient department at King Abdullah University Hospital (KAUH) in Irbid, Jordan.

### 3.2 Participants

The target population for this study was persons with type 2 diabetes. All adult males and females who were diagnosed with T2DM at least 3 months before the study, were invited. Further eligibility criteria included being prescribed medications for diabetes, with or without comorbidity, and in the implementation stage. The implementation stage of medication adherence is the stage where an individual has started taking medications as prescribed (Vrijens et al., 2012; Ribaut et al., 2020). Besides, patients who were admitted to a hospital, had acute illnesses, were pregnant or had planned pregnancy, were breastfeeding, and so on, were not eligible. Moreover, those who did not consent to participate were excluded.

### 3.3 Sample size

The suggested sample size for factor analysis ranges from 3—20 times the number of variables, while the absolute sample size ranges from 100—1000 (Mundfrom et al., 2005). A convenience sampling method was employed to select 100–150 participants. This was based on the findings of Muthén and Muthén that confirmatory factor analysis (CFA) with normally distributed and no missing data may yield a power of 0.8 (Muthén and Muthén, 2002).

### 3.4 Research instrument

The Arabic version of the General Medication Adherence Scale (GMAS) was used with permission (Naqvi et al., 2020a). The scale consisted of 11 items that measured adherence to medications. Each item had 4 possible responses, and each response had a score. Participants were asked to select the item that best described their medicine-taking practice. The scores from all individual items were summed up to report a cumulative adherence score. The maximum achievable adherence score was 33, and was categorized into categories; poor (0—10), low (11—16), partial (17—26), good (27—29), and high (30—33) adherence (Naqvi et al., 2020a). Additionally, the score could be categorized as dichotomous, i.e., adherent (≥27) and non-adherent (≤26). The detailed scoring of the scale is described previously (Naqvi et al., 2020a; Naqvi et al., 2020b).

### 3.5 Statistical analysis

The model fitness was evaluated by confirmatory factor analysis (CFA). It was conducted with the formation of a structural equation model for a three-factor model. The 3-factor model was considered based on the previous validation of the Arabic version among patients in Sudan and Saudi Arabia (Naqvi et al., 2020a; Mahmoud et al., 2021). Fit indices, namely, goodness of fit index (GFI), Tucker Lewis index (TLI), comparative fit index (CFI), and root mean square error of approximation (RMSEA) were observed. The factor validity was established if the fit indices were in the acceptable range, i.e., GFI, AGFI, TLI, CFI >0.9, and RMSEA <0.08.

In addition, the construct validity was assessed. measured by assessing the corrected item-total correlation. This assessment was carried out by adopting the methods used by Raharjanti and others to assess construct validity (Raharjanti et al., 2022). Construct validity is the extent to which an assessment measures a theoretical concept it is expected to measure (Hajjar, 2018). The item total correlation (ITC) was analysed. An ITC between 0.3 and 0.49 was considered moderate while ITC >0.5 was considered strong (Hajjar, 2018).

Further, the reliability was assessed using Cronbach’s alpha and was considered satisfactory if it was >0.7 (Sarmento and Costa, 2019). Also, item-deletion was carried out to review the contribution of each item towards internal consistency of scale.

### 3.6 Data collection and reporting

An informed written consent form was sought from participants before handing the survey. Participants who returned signed consent forms were provided with the questionnaire. Participants were informed that participation was voluntary, and their decision to participate will not have any impact on the healthcare they receive. The questionnaire had no personal identifiers and were stored separately to the consent forms. The data was collected once from a participant at the venue. Patients who were eligible at the hospital were approached for recruitment. The questionnaire was handed to the participant for self-administration and was collected later. It was without any personal identifiers, and hence, it could not be linked to the participants.

The data initially gathered was coded in Microsoft Excel. Later, it was imported and analysed using IBM SPSS version 23 (Armonk, NY). CFA was conducted using IBM AMOS version 25. The data was digitized and verified. Categorical data were expressed in sample count (n) and percentage (%), while continuous data were expressed in mean (X) and standard deviation (SD).

### 3.7 Ethics statement and informed consent

The study was approved by the Institutional Review Board of Jordan University of Science and Technology (Ref: 59/117/2018). Participants were informed that the study participation was voluntary and their decision to participate would not have any impact on the healthcare they receive. The questionnaires had no personal identifiers and were stored separately to the consent forms.

## 4 Results

A total of 119 completed and useable surveys were received.

### 4.1 Participants’ characteristics

The mean age of participants was 56.4 (±15.07) years. Most participants were above 50 years (n = 80, 67.2%), identified as female (n = 70, 58.8%), and indicated their status as married (n = 89, 74.8%). Most participants were graduates (n = 47, 39.5%) and had a monthly income between JOD 201—500 (n = 41, 34.5%). Majority of participants were prescribed up to 2 medications (n = 108, 90.2%), had comorbidities (n = 65, 54.6%), and received medicines by government supply (n = 57, 47.9%).

The mean Hb_A1c_ was 7.65 (±1.34) % (95% CI, 7.41%—7.9%). The mean random blood glucose was reported at 185.4 (±71.5) mg/dL (95% CI: 172.5 mg/dL—198.5 mg/dL). Of those (n = 65, 54.6%) who indicated having comorbidities, 40 patients had single comorbidity, i.e., hypertension (HTN), asthma (n = 1), gout (n = 1), kidney disease (n = 1), irritable bowel syndrome (n = 1), ulcer (n = 1), dyslipidaemia (n = 1), thyroid disease (n = 1), previous heart failure (HF) (n = 1), and neck and back pain (n = 2). Fifteen patients had multimorbidity, i.e., HTN and arthritis (n = 1), HTN and kidney disease (n = 1), gout and arthritis (n = 1), HTN and prostate enlargement (n = 1), dyslipidaemia and previous HF (n = 1), HTN and osteoporosis (n = 1), asthma and arthritis (n = 1), HTN and dyslipidaemia (n = 3), HTN and a previous case of heart failure (HF) (n = 3), HTN, asthma and rheumatoid arthritis (RA) (n = 1), HTN, gout and dyslipidaemia (n = 1). The mean adherence score was 27.5 (±6) and ranged from 6 to 33. About two-third of the sample were adherent to their therapy (n = 79, 66.4%) (Table 1).

**TABLE 1:** Sociodemographic and adherence characteristics of participants (n = 119).

Characteristics	Frequency (n)	Percentage (%)
**Age**		
≤50 years	39	32.8
>50 years	80	67.2
**Gender**		
Male	49	41.2
Female	70	58.8
**Marital status**		
Single	12	10.1
Married	89	74.8
Other	18	15.1
**Education**		
Primary	25	21
Secondary	21	17.6
Intermediate	26	21.8
Graduate	47	39.5
Monthly income (USD)*		
JOD ≤200 (USD ≤282.09)	24	20.2
JOD 201—500 (USD 283.5–705.23)	41	34.5
JOD 501—700 (USD 706.64–987.32)	30	25.2
JOD 701—1000 (USD 988.73–1410.45)	13	10.9
JOD >1000 (USD >1410.45)	11	9.2
**Comorbidities**		
Yes	65	54.6
No	54	45.4
**Number of Medicine taken**		
≤2	108	90.8
>2	11	9.2
**Mode of obtaining most medicines**		
Government supply	57	47.9
Insurance	23	19.3
Out of pocket	39	32.8
**Adherence status**		
Non-adherent to therapy	40	33.6
Adherent to therapy	79	66.4

*1 Jordanian Dinar (JOD) equals United States Dollar (USD) 1.41 at the time of this writing.

### 4.2 Validation results of GMAS-AR

#### 4.2.1 Model fitness using confirmatory factor analysis

**FIGURE 1:**
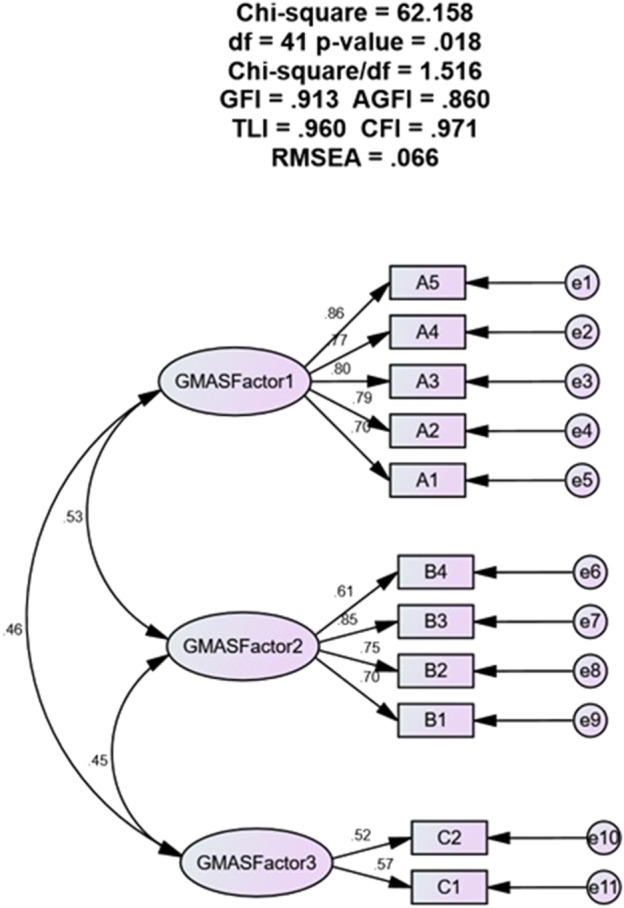
Structural equation model.

The Figure 1 shows the results of a confirmatory factor analysis (CFA) of GMAS. The CFA results showed that a 3-factor model of the GMAS had a good fit to the data, with all of the fit indices within acceptable ranges. The three factors were: Factor 1: Behaviour related non-adherence, Factor 2: Comorbidity and medication related non-adherence, Factor 3: Cost-related non-adherence. The factor loadings for the 3-factor model were all significant, with most of the loadings being in the moderate to strong range. This suggests that the 3-factor model is a good representation of the underlying structure of the GMAS. CFA analysis together with SEM modelling for a 3—factor structure showed a model fit with fit indices in acceptable ranges. The values were as follows: χ^2^ = 62.158, df = 41, *p* = 0.018, χ^2^/df = 1.516, GFI = 0.913, AGFI = 0.860, TLI = 0.960, CFI = 0.971 and RMSEA = 0.066.

#### 4.2.2 Construct validity

The corrected ITC values for each item of the questionnaire were analysed. The highest value for corrected item-total correlation ITC was 0.807 while the lowest value was 0.275.10 out of 11 items had corrected ITC values >0.5 indicating a strong contribution towards overall medication adherence. All values were positive (+). One item (item 11) had a corrected ITC value of 0.275 which was <0.3. Item 11 of the questionnaire assessed cost-related non-adherence, a distinct facet of adherence that is differentiated from patient behaviours, comorbidities, and pill burden. Therefore, it was retained. The scale was considered to have construct validity.

### 4.3 Reliability results of GMAS-AR

The overall reliability (Cronbach’s α) of the scale (n = 11) was 0.907. The intraclass correlation coefficient was reported at 0.907 (95% CI, 0.880—0.930). The reliability of the scale remained between 0.89—0.92 during item deletion (Table 2).

**TABLE 2:** Reliability and internal consistency.

GMAS items	Corrected ITC	α if item deleted
1	0.658	0.898
2	0.744	0.893
3	0.751	0.893
4	0.736	0.894
5	0.807	0.890
6	0.638	0.900
7	0.719	0.895
8	0.792	0.891
9	0.575	0.903
10	0.523	0.905
11	0.275	0.920

## 5 Discussion

A study by Awwad and others highlighted that most Jordanian patients had low adherence. In addition, it was highlighted in the study that education, monthly income, and knowledge of patients impact their adherence to medications (Awwad et al., 2015). Moreover, Basheti and others reported non-adherence in slightly less than half of their study population. Further, they establish a relationship between adherence score and comorbidity, number of medicines, etc. (Basheti et al., 2016). The current study reported a large proportion of patients to be adherent. However, due to the limited number of responses collected, it cannot be definitively confirmed if this outcome occurred by chance or represents a true pattern.

The validation process included several statistical approaches, such as CFA with SEM and evaluation of internal consistency through Cronbach’s (α), ITC and α based on item deletion. The values for fit indices excluding RMSEA obtained in this study were >0.95 for TLI and CFI, >0.9 for GFI, and <0.9 for AGFI. Usually, a value >0.95 is considered excellent, while a value >0.9 is considered acceptable (Sarmento and Costa, 2019). In addition, the value for RMSEA obtained in this study was <0.08. Though a smaller value, such as < 0.06, is appreciable for RMSEA, studies have provided 0.08 as a cut-off criterion as well (Sarmento and Costa, 2019). The GFI obtained in this study was 0.913 while AGFI was 0.860. Available evidence report that GFI and AGFI are considered as good if the value is close to 1.0. Also, the AGFI is always less than or equal to the GFI. Thus, based on the current literature the values for both were considered acceptable (Hayashi et al., 2011). In addition, the χ^2^/df value obtained for this study was between one to two which is considered as good (Sarmento and Costa, 2019).

In comparison, the Mahmood and others in their validation study involving GMAS-AR in patients with diabetes in Sudan reported a χ^2^/df value >3, and CFI, TLI, GFI and AGFI >0.9, while RMSEA was >0.073. The value for Cronbach’s α was 0.834 (Mahmoud et al., 2021). In addition, another study was conducted in Saudi Arabia in which Arabic version of GMAS was validated in patients with chronic ailments, reported a value >0.95 for all fit indices and <0.06 for RMSEA. A Cronbach’s α value of 0.865 was reported (Naqvi et al., 2020a). In this study, the value Cronbach’s α was reported at 0.907. Therefore, despite being small-scale compared to its predecessors, the results aligned with the previous two studies (Naqvi et al., 2020a; Mahmoud et al., 2021).

The CFA results provide support for the validity of the Arabic version of the GMAS in the studied population. The good fit of the model suggests that the scale adequately measures adherence based on the underlying constructs it is expected to measure. The factor loadings also substantiate that ability of the scale to measure adherence based on the expected factor structure. In addition, the item-total correlation values were positive and ranged from 0.275 to 0.807 with majority of items having corrected ITC >0.5. This highlighted that each item in the questionnaire had a contributed positively towards measuring medication adherence and the scale had construct validity.

The availability of this scale for use in Jordanian patients would help clinicians to evaluate and monitor the pharmacotherapy of patients and would set an example for further investigation pertaining to the reliability and validity of GMAS in other disease populations in Jordan. The element of costing distinguishes this scale from the available instruments. It was observed in this study that slightly more than a third of patients paid for the medications out-of-pocket. Hence, this scale would enable the clinicians to evaluate the non-adherence due to the cost of medications as well.

The study was based on convenience sampling and was carried out in a single hospital. Besides, the number of surveys obtained was low. Based on these factors, some limitations of this study are identified. These aspects would affect the generalizability of results and may not represent the entire population. The risk of sampling error cannot be ruled out. This study may have low statistical power and may not be able to detect small and meaningful association between participants’ characteristics and their adherence score. As a result, sub-group analyses and multivariate regression analyses highlighting determinants of adherence could not be conducted on this dataset. Nevertheless, it satisfied the sampling requirements based on the statistical parameters for CFA analysis. However, the results pertaining to medication adherence and clinical information should not be generalized.

## 6 Conclusion

The results of this study highlight that the Arabic version of the GMAS is a reliable and valid tool to assess the medication adherence of patients with T2DM in Jordan. A new scale is now available for documenting adherence to medications in this population. This study also serves as a model for further validation the scale in other patient populations.

## Data Availability

The original contributions presented in the study are included in the article/Supplementary Material, further inquiries can be directed to the corresponding author.
